# Temperature dependence of the microwave dielectric properties of $\gamma$-aminobutyric acid

**DOI:** 10.1038/s41598-021-97178-7

**Published:** 2021-09-10

**Authors:** Jie Hou, Sisay Mebre Abie, Runar Strand-Amundsen, Yuri M. Galperin, Joakim Bergli, Christin Schuelke, Sina Hashemizadeh, Ørjan Grøttem Martinsen

**Affiliations:** 1 Department of Physics, University of Oslo, Sem Sælands vei 24, 0371 Oslo, Norway; 2 Department of Clinical and Biomedical Engineering, Oslo University Hospital, Sognsvannsveien 20, 0372 Oslo, Norway; 3 Foundation for Research on Information Technologies in Society (IT’IS), Zeughausstrasse 43, 8004 Zurich, Switzerland; 4 A. F. Ioffe Physico-Technical Institute of Russian Academy of Sciences, Polytekhnicheskaya 26, St. Petersburg, Russia 194021

**Keywords:** Neurology, Engineering, Physics

## Abstract

The GABA molecule is the major inhibitory neurotransmitter in the mammalian central nervous system. Through binding to post-synaptic neurons, GABA reduces the neuronal excitability by hyperpolarization. Correct binding between the GABA molecules and its receptors relies on molecular recognition. Earlier studies suggest that recognition is determined by the geometries of the molecule and its receptor. We employed dielectric relaxation spectroscopy (DRS) to study the conformation and dielectric properties of the GABA molecule under physiologically relevant laboratory conditions. The dielectric properties of GABA investigated have given us new insights about the GABA molecule, such as how they interact with each other and with water molecules at different temperatures (22°C and 37.5°C). Higher temperature leads to lower viscosity, thus lower relaxation time. The change in the GABA relaxation time due to concentration change is more associated with the solution viscosity than with the GABA dipole moment. A resonance behavior was observed with high GABA concentrations at physiological temperature, where there might be a phase transition at a certain temperature for a given GABA concentration that leads to a sudden change of the dielectric properties.

## Introduction

GABA is an important inhibitory neurotransmitter, present in 25–50% of the brain’s synapses and has a critical role in regulating excitability throughout the brain^1^. In the adult brain, GABA mediates its inhibitory effect by hyperpolarizing the membrane and by shutting down the excitatory inputs^2^. This prevents the neurons from reaching the threshold of an action potential, hindering the release of neurotransmitters. Ultimately, GABA reduces the activity of the neurons to which it binds, thereby calming down the nervous activity in the brain.

The GABA molecule can exist in different conformations as it has a flexible carbon backbone and different conformations will lead to different behaviors^3^. Thus, an understanding of the nature and stability of various conformations of GABA in a liquid mixture environment is very important as its binding to different receptors occurs in different conformations^4^. There are a series of neurodegenerative diseases that are related to the GABA function in the brain, such as epilepsy, Huntington’s diseases and Alzheimer’s disease^5^.

By discovering the dielectric properties of the GABA molecule in the human brain we may understand the mechanisms of these diseases better, and the knowledge might be used to develop new strategies to combat these diseases. With known GABA conformation, we may be able to investigate whether the condition of the patient is due to incorrect binding or if it is due to a deficiency of the GABAergic neurons so that they are not able to produce sufficient levels of GABA. Studying the GABA molecule in a physiological temperature range can bring us closer to clinical investigations on patients with neurodegenerative or neurological diseases that are related to the GABA neurotransmitter.

GABA exists both in animals and plants^6^ and previous studies investigated the GABA dielectric properties at room temperature^4,7–9^, which is not closely relevant in the case of human brains. Kaatze et al.^8^ studied the complex permittivity of GABA in aqueous solutions and found that the solute relaxation time is almost independent of its dipole moment, which rises the following question: What is the underlying cause of the change in relaxation time? Ottosson et al.^7^ investigated the conformation of the GABA molecule in liquid water. They found that GABA adopts a nearly linear, unfolded conformation in aqueous solution at room temperature, which rises the question: How will the GABA conformation change when we increase the temperature? Shitaka et al.^4^ investigated the dielectric features of GABA for molecular recognition by receptors. They discovered that the receptors will detect differences in magnitude and direction of the dipole moment of different neurotransmitters, thus allowing correct molecular recognition. This implies that the dipole moment is of significant importance in molecular recognition. However, to the best of our knowledge, there have not been any studies of the dielectric properties of GABA at physiological temperature.

The dipole structure of the GABA molecule allows us to study its dielectric properties, since a dipole molecule will reorient in response to an applied electric field. The DRS is based on the interaction of an external electric field with the dipole molecules in the sample, and it was used to obtain the permittivity data of GABA solutions. We compare the GABA dielectric properties at room and physiological temperature to investigate the following issues:

1. How will the temperature affect the complex permittivity spectra of different GABA concentrations?
2. How will the GABA conformation change when we increase the temperature?
3. What is the underlying cause of the change in GABA relaxation time?
4. Is the relative contribution of water to the total dielectric behaviour dependent on the temperature?
5. Will temperature affect how GABA molecules interact with each other and with the water molecules?

## Results and discussions

### Dielectric spectra

With the purpose of determining the dielectric properties of the GABA molecule, a traditional relaxation model based on a superposition of two Cole-Cole equations (1) was used to fit the measured permittivity spectra:

1 $$\begin{aligned} \epsilon ^*(\omega ) = \epsilon _{\infty } + \frac{\Delta \epsilon _1}{1+(i\omega \tau _1)^{1-\alpha _1}} + \frac{\Delta \epsilon _2}{1+(i\omega \tau _2)^{1-\alpha _2}}. \end{aligned}$$

A superposition of two Cole-Cole equations was applied to take account for both the GABA and the water molecules. The relaxation strength is proportional to the area under the dielectric loss peak. $\Delta \epsilon _1 = \epsilon _{\text {s}} - \epsilon _1$ and $\Delta \epsilon _2 = \epsilon _1 - \epsilon _\infty$ are the relaxation strengths for GABA and water, respectively, and $\tau _1$ and $\tau _2$ are the corresponding relaxation times. *i* is the imaginary unit. The parameters $\epsilon _\infty$, $\tau _1$, $\tau _2$, $\Delta \epsilon _1$, $\Delta \epsilon _2$, $\alpha _1$, $\alpha _2$ can be obtained by fitting the Eq. (1) to the experimental permittivity data for each of the samples. Two sets of parameters were extracted; the contribution of the GABA molecules to the total dielectric behavior of the solution, and the contribution of the water molecules. $\epsilon _\infty$ is a joint parameter for both GABA and water molecules and indicates the high frequency limit of the permittivity. The obtained values for the fitting parameters $\alpha _1$ and $\alpha _2$ were lower than 0.2, and most of the $\alpha _1$ values were around 0.02.

**Figure 1:**
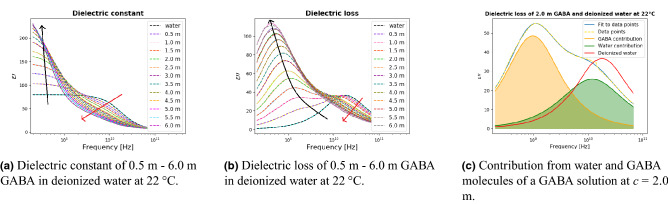
Real ($\epsilon '$) and imaginary ($\epsilon ''$) parts of the complex permittivity of GABA solutions and pure deionized water together with the fitted permittivity spectra as a function of frequency, at 22 °C. GABA concentration ranges from 0.5 to 6.0 m with a 0.5 m step length between each of the concentrations. Dashed lines represent the experimental data points and solid lines represent the fit to the relaxation model (1). Figure 1c, the solid blue curve represents a non-linear square fit to the experimental dielectric loss data points, and the dashed yellow curve shows the experimental data points. In addition, the red solid curve represents the dielectric loss spectrum for pure deionized water. Dielectric measurements were performed in a frequency range 200 MHz–67 GHz.

Typically, the main contribution of the dielectric relaxation of bulk water in this frequency region is well described by using the Debye function. However, when mixing water with other substances, a possible broadening could appear in the spectra. Therefore, a symmetric Cole-Cole function was used where the second Cole-Cole function accounted for the GABA molecules relaxation. Figure 1 shows the permittivity spectra of GABA at 0.5 molal–6.0 molal (m) of GABA dissolved in deionized water at room temperature (22 °C). Figure 1a shows the dielectric constant $\epsilon '$ spectra which is the in-phase contribution to the total polarization of the GABA solution, while Fig. 1b shows the corresponding out-of-phase contribution, $\epsilon ''$. For comparison, the permittivity spectrum of deionized water measured at the same temperature has been added to the subfigures. As shown in Fig. 1a, all samples exhibited a relatively high dielectric constant at low frequencies (200 MHz–1 GHz), and then decreased with the increasing frequency. The addition of GABA to the deionized water led to an increase in the dielectric response. This observation agrees well with the observations reported by Ottosson et al.^7^ and indicates that the process at the lower frequency range depends on the GABA concentration, and it is likely dominated by the GABA relaxation process. This observation is furthermore strengthened by the fact that the GABA molecule is larger than the water molecule and should hence give a characteristic frequency that is lower than for the water molecule.

The increase in the rate of the dielectric constant (compared to the dielectric constant of the previous GABA concentration) is reduced with increasing GABA concentration at the lower frequency range. In the middle frequency range (1–10 GHz), we observe that the dielectric constant decreases and shifts towards the lower frequency range, with increasing GABA concentration, as the red arrow indicates in Fig. 1a. However, this change of the dielectric constant in the intermediate frequency range was less significant compared to the change at low frequencies, meaning that the process at middle to high frequency range is less dependent on the GABA concentration. Therefore, it is most likely related to the relaxation process of the water molecules.

With regards to the dielectric loss presented in Fig. 1b, as we increase the GABA concentration, the relaxation peak of the dielectric loss curve shifts to the lower frequency range, along with a significant increase of the dielectric loss amplitude at the lower-middle frequency range. This indicates that the addition of the GABA molecules to the solution leads to a higher energy loss. Simultaneously, a decrease in the dielectric loss at a higher frequency range was found. Since the relaxation mechanism at the higher frequency range is related to the water molecules, it is thus associated with the decreasing water molecule proportion, as more GABA molecules were added into the solution.

The measured dielectric loss spectra show two dispersion processes with two peaks well separated from each other, as displayed in Fig. 1b. A $\gamma$-dispersion was observed for all concentrations. A clearer view of the two dispersions is shown in Fig. 1c. It illustrates the decomposition of the $\epsilon ^{\prime \prime }$ of a 2.0 m GABA solution into the contribution of the solute GABA and the solvent water. Two dispersions can be seen in Fig. 1c. The first dispersion (orange curve) in the lower frequency range is due to the relaxation mechanism of the solute GABA molecules. GABA molecules are much bigger than water molecules ($V_{\text {m}}$ for water^9^ is 30 $\times$ $10^{-30}$ ${\text {m}}^3$ , whereas $V_{\text {m}}$ for GABA^10^ is 121.60 $\times$ $10^{-30}$ ${\text {m}}^3$), and thus the time needed for GABA molecules to rotate is much longer than for water molecules. Therefore, the GABA contribution dominates in the lower frequency range. The second dispersion (green curve) is due to the dielectric relaxation of the solvent water molecules. Comparing the second dispersion (green curve) with the dielectric loss spectrum of the pure deionized water (red curve), we observe a slight shift to the lower frequency range. This indicates that the solvent water molecules were perturbed in their orientational motions by the presence of the solute GABA molecules. This observation is accordant with the findings of the study carried out by Ottosson et al.^7^. When comparing the dielectric loss spectrum of deionized water and the water contribution to the total dielectric loss spectrum of a GABA solution, we observe a broadening of the dielectric loss peak when GABA is present. This result confirms the hypothesis by Levy et al.^11^, who stated that whenever a water molecule interacts with another dipolar molecule, a broadening of the dielectric relaxation peak occurs.

**Figure 2:**
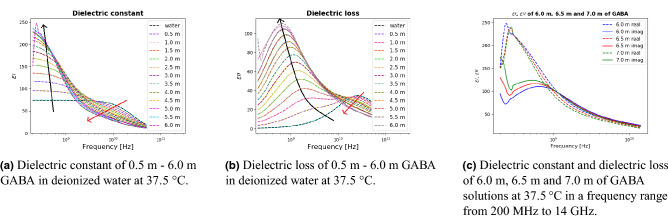
Real ($\epsilon ^\prime$) and imaginary ($\epsilon ^{\prime \prime }$) parts of complex permittivity of GABA solutions and deionized water together with the fitted permittivity spectra as a function of frequency, at 37.5$^{\circ }C$. GABA concentration ranges from 0.5 to 6.0 m with a 0.5 m step length between each of the concentrations. Dashed lines represent the experimental data points and solid lines represent the fitting to the relaxation model. Dielectric measurements were performed in a frequency range from 200 MHz–67 GHz.

To explore the temperature dependence of a GABA solution, we measured permittivities for the same GABA concentrations at 37.5$^{\circ }C$. Figure 2 illustrates similar permittivity spectra at 37.5$^{\circ }C$ for most of the GABA concentrations, when compared to the permittivity spectra at 22$^{\circ }C$ as shown in Fig. 1. However, when the temperature increased from 22$^{\circ }C$ to 37.5$^{\circ }C$, the 6.0 m GABA solution exhibited an unusual spectrum. This sudden change occurred when the temperature increased from 29$^{\circ }C$ to 30$^{\circ }C$. In order to further investigate this behavior, 6.5 m and 7.0 m GABA solutions were measured for comparison.

The sudden change of the permittivity spectra occurred at different temperatures for different concentrations. We found that the temperature needed for this change to occur decreased with increasing GABA concentrations, as shown in Table 1. To the best of our knowledge, this behavior of the permittivity spectra of the GABA molecule has not been reported before. The reason for such a behavior is not fully clear. Both the intermolecular and the intramolecular forces can possibly be responsible for this behavior^9,11–15^. Changes in concentration and solution temperature may lead to changes in the intermolecular force between GABA-GABA dipoles and GABA-water dipoles and in the intramolecular force that holds the atoms together within a molecule. Moreover, there might be a phase transition at a certain temperature for each of the GABA concentrations that leads to this change. We hope that this experimental finding will attract attention of specialists on microscopic numerical calculations of thermodynamics of solutions. To make sure that the observed phenomena are not stimulated by the measuring AC field we have confirmed that the dielectric spectra do not depend on the field amplitude.

**Table 1:** Temperatures of sudden change in the permittivity spectra for different concentrations of GABA.

Concentration (m)	Temperature ($^\circ$C)
6.0	29.35
6.5	28.43
7.0	27.12

For the two cases investigated (22$^{\circ }C$ and 37.5$^{\circ }C$), the increasing rate of the dielectric constant decreases with the GABA concentration at the lower frequency range. For each GABA concentration, we calculated the change in dielectric constant relative to the previous GABA concentration divided by the concentration $\Delta = (\epsilon ' - \epsilon '_{\text {w}})/c$, where $\epsilon '$ is the dielectric constant of the GABA solution, $\epsilon '_{\text {w}}$ is the dielectric constant of water ($\epsilon '_{\text {w, 22}{^{\circ }C}}$ = 79.5, $\epsilon '_{\text {w, 37.5}{^{\circ }C}}$ = 73.6, both are experimentally obtained.) and *c* is the concentration. We noticed that the overall $\Delta$ values were lower at 37.5$^{\circ }C$ at low concentrations and $\Delta$ values were similar for both cases at high concentrations, except for 6.0 m GABA in deionized water at 37.5$^{\circ }C$, due to the significant change in the dielectric behavior at low frequencies.

### Relaxation time

For both cases, the relaxation time increased almost linearly with increasing GABA concentration, which agrees with our observations presented in Figs. 1b and 2b. The overall relaxation time for GABA in deionized water at 22$^{\circ }C$ was much higher than the relaxation times at 37.5$^{\circ }C$. It increased from 96.46 to 369.00 ps at 22$^{\circ }C$ and increased from 66.52 to 212.68 ps at 37.5$^{\circ }C$. We think that this is because higher temperatures allow faster molecular motions.

Using the derived relaxation times, we found the GABA relaxation time at the infinite dilution, $\tau _{1}^0$, by taking the intercept of a linear regression of the extracted relaxation time data points. By using the modified Stokes-Einstein-Debye equation^9^ we relate the microscopic relaxation time, $\tau _1^\prime$, to the macroscopic dielectric relaxation time, $\tau _1$ (measured time), and in this way connect the relaxation time with the volume of the GABA molecules. Both relaxation times are proportional to the effective volume of rotation, $V_{\mathrm {eff}}$, and to the viscosity, $\eta$:

2 $$\begin{aligned} \tau _1' = \frac{2\epsilon _{\text {s}} + \epsilon _{\infty , 1}}{3\epsilon _{\text {s}}}\, \tau _1 = \frac{3V_{\text {eff}}\eta }{k_{\text {B}} T}. \end{aligned}$$

Here $\epsilon _{\infty , 1}$ is the high-frequency limit of the *j* = 1 solute process. At the infinite dilution limit, the viscosity will approach the viscosity of pure water $\eta \rightarrow \eta _{\text {w}} \approx 9.55 \times 10^{-4}$ Pa s at 22$^{\circ }C$ and $6.95 \times 10^{-4}$ Pa s at 37.5$^{\circ }C$, in addition to $\epsilon _{\infty , 1} \rightarrow \epsilon _{\text {s}}$ and $\tau '_1 \rightarrow \tau$^9^. In the infinite dilution limit, we have:

3 $$\begin{aligned} \tau ^0_1 = \frac{3V_{\text {eff}}\eta _{\text {w}}}{k_{\text {B}} T}. \end{aligned}$$

We used the relaxation time of GABA at infinite dilution limit $\tau ^0_1$ and the water viscosity $\eta _{\text {w}}$ to calculate the effective rotational volume $V_{\text {eff}}$. With known $V_{\text {eff}}$ and the volume of GABA molecule $V_{\text {m}}$, the product ($f_{\text {s}} C$)^9^ can be found by using equation:

4 $$\begin{aligned} V_{\text {eff}} = f_{\text {s}}CV_{\text {m}}. \end{aligned}$$

Here $f_{\text {s}}$ is a pure geometrical dimensionless parameter, which accounts for the deviation of the molecule shape from the ideal spherical form, and it can be calculated from the molecule geometry^9^. *C* is an experimentally determined dimensionless parameter which is a measure of the coupling between the rotating molecule and its surroundings^16^. As Table 2 shows, for both cases, the effective rotational volumes are smaller than the actual volume of the GABA molecule, and the effective volume decreased with increasing temperature. As for the ($f_{\text {s}} C$) product of GABA in deionized water, the value of the product ($f_{\text {s}} C$) decreased from 0.7 to 0.55 when the temperature increased from 22$^{\circ }C$ to 37.5$^{\circ }C$.

**Table 2:** Relaxation times of GABA at the infinite dilution under two conditions: GABA dissolved in deionized water at 22$^{\circ }C$ and 37.5$^{\circ }C$.

Parameter	$W_{22^{\circ }C}$	$W_{37.5^{\circ }C}$
$\tau _{1}^0$ (ps)	59.61	47.45
$V_{\text {m}}$ ($10^{-30} {\text {m}}^3$)	121.60	121.60
$V_{\text {eff}}$ ($10^{-30} {\text {m}}^3$)	84.75	67.46
$f_{\text {s}} C$	0.70	0.55

Table shows the calculated effective rotational volume, $V_{\text {eff}}$, the calculated ($f_{\text {s}} C$) product which accounts for deviations of the GABA molecule from a spherical shape and the coupling between GABA molecule and its surroundings. We used the experimental GABA molecular volume ($V_{\text {m}}$) value as described by Sirimulla et al.^10^, expressed as volume per molecule.

### Relaxation strength

**Figure 3:**
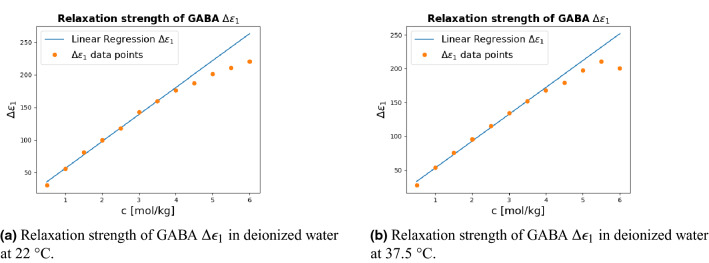
Relaxation strength $\Delta \epsilon _1$ of GABA molecules as a function of GABA molal concentration *c* at 22$^{\circ }C$ and 37.5$^{\circ }C$. Solid lines correspond to linear regressions obtained by considering only the lower concentration data points, $c \le$ 4.0 m.

Figure 3 shows the GABA relaxation strength as a function of the GABA concentration.

Up until *c* = 4.0 m, the relaxation strength $\Delta \epsilon _1$ is linearily proportional to the GABA concentration, but for higher concentrations the linear relationship does not hold. This may be related to the GABA-GABA dipole antiparallel alignments which can lead to a slight aggregations of the GABA molecules. The stronger GABA-water interactions when the proportion of the GABA molecule increases may be another reason for this nonlinear behavior, and it indicates that the GABA molecules have fairly free rotations from other GABA molecules up to $c \le$ 4.0 m.

### Dipole moment

There are different GABA receptors in the human brain, and these receptors need to recognize and bind to the GABA molecule in order to express an inhibitory effect in the neurons. The receptors detect the differences in the magnitude and the direction of the GABA dipole moment, and thus allow correct molecular recognition^4^. Since GABA is a neutral zwitterion at a neutral pH value, it maintains a well defined dipole moment with a large magnitude in aqueous solution. Therefore, the GABA relaxation is considered in this work to be completely due to the rotational movements of the GABA molecules. The following equation was used to calculate the dipole moment of the GABA molecules,

5 $$\begin{aligned} \mu _{\text {eff, }j} = \sqrt{\frac{\Delta \epsilon _{j} \cdot 3[\epsilon _{\text {s}}+(1-\epsilon _{\text {s}})A_{j}]}{\epsilon _{\text {s}}} \cdot \frac{k_{\text {B}}T\epsilon _0c_{j}}{N_{\text {A}}}}. \end{aligned}$$

Here $\mu _{\text {eff, }j}$ is the effective dipole moment for* j*th relaxation process, $\epsilon _{\text {s}}$ is the static permittivity, $\epsilon _0$ is the permittivity of vacuum, $N_{\text {A}}$ is the Avogadro’s number, $k_{\text {B}}$ is the Boltzmann constant and *T* is the temperature in Kelvins. This equation connects the relaxation strength $\Delta \epsilon _{j} = \epsilon _{j} - \epsilon _{j+1}$ of relaxation process *j* to the molar concentration of dipolar substances, $c_{j}$ and their dipole moments, $\mu _{\text {eff, }j}$^17^. The shape factor $A_{j}$ accounts for the shape of the molecule, $A_{j} = 1/3$ for spheres^18,19^ was used for the calculations as no values for GABA was found.

The calculated electric dipole moment of GABA varied from 19.93 to 25.55 D at 22$^{\circ }C$ and 19.50 D to 25.06 D at 37.5$^{\circ }C$. There was no significant difference between the two cases, which indicates that the substantial change in the relaxation times of the GABA molecules is almost independent of the GABA dipole moment. This agrees with the results reported by Kaatze et al.^8^ and Rodríguez-Arteche et al.^9^. This leads us to believe that the increasing relaxation time with increasing concentration is not predominantly due to the GABA molecule shape change, which is closely related to the electric dipole moment, but rather more related to the interactions between the neighboring dipole molecules. Kaatze et al.^8^ proposed that the association of the GABA molecules with partly antiparallel arrangement of the dipole moment might be expected at high concentrations of GABA. This expectation is in agreement with our observations on the empirical Kirkwood correlation factor $g_{j}$, which is a measure of the strength of the orientational correlations between molecule dipoles and its nearest neighboring dipoles. It describes the short-range dipole-dipole self-correlation^20^. $g_{j} = 1$ implies a statistical arrangement of dipoles, $g_{j} > 1$ indicates a tendency towards parallel alignment and $g_{j} < 1$ means a tendency towards antiparallel alignment^21,22^. We observed that with increasing GABA concentrations, the antiparallel arrangement is strengthened.

At infinite dilution, the dipole moment $\mu _{\text {GABA}}^0$ extracted from a linear regression was 25.5 D, which is in agreement with the reported value of 25.8 ± 0.3 D by Ottosson et al.^7^. The measured dipole moment at 1.0 m GABA at 22$^{\circ }C$ was 24.69 D. The dipole moment for 1.0 M GABA has previously been reported by three different groups to be 20.4 D^8^, and 25.5 D for 1.0 m GABA^7^ and the simulated value^23^ from the VEGA ZZ software 22.11 D. Considering the different temperature and frequency range used, the difference appears to be insignificant. Moreover, Odai et al.^24^ simulated the electric dipole moment of GABA in vacuum to be 21.98 D. This value is very close to our calculated dipole moment value and indicates that the structure of GABA in vacuum is very similar to that in an aqueous solution. As for 37.5$^{\circ }C$, to the best of our knowledge, we are the first to study the GABA molecules at this temperature; hence, there was no published work to compare with.

**Figure 4:**
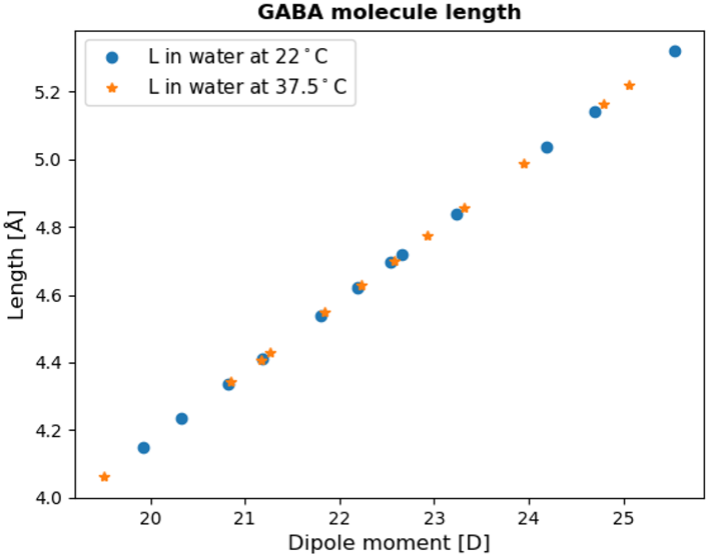
Calculated distance between the two charged groups of a GABA molecule for different concentrations of GABA in deionized water at 22$^{\circ }C$ and 37.5$^{\circ }C$. The X-axis represents the dipole moment $\mu$ in D (Debye), the Y-axis represents the distance *L* in Å (Ångstrøm).

In general, the distance *L* between the center of the anionic and cationic group of a GABA molecule can be calculated using the equation $\mu = qL$, where $\mu$ is the dipole moment and *q* is the elementary charge, $q = 1.602\times 10^{-19}$ C. The distance *L* gives a good indication on the conformation of the GABA molecule. The theoretical distance of the anionic and cationic group of a GABA molecule was estimated using equation

6 $$\begin{aligned} L = L_0\sqrt{2m-1.5\left( 1-3^{-m}\right) }. \end{aligned}$$

with $m = 5$ for GABA molecule was 4.49 Å^8^. Comparing this theoretical distance with the experimental distance between the positively charged and the negatively charged group of the GABA molecule shown in Fig. 4, the theoretical distance obtained is slightly shorter than that of the experimental distance with the largest dipole moment (lowest GABA concentration). The theoretical distance between the two charged groups being similar to the experimental distance indicates that the GABA molecule has an extended conformation with the positive and negative charged parts at either end, and this conformation stays nearly constant for both cases investigated. Our result for GABA in deionized water at 22$^{\circ }C$ agreed well with the results reported by Odai et al.^24^ and Ottosson et al.^7^.

Knowing the dipole moment of the GABA molecule enabled us to investigate the GABA-GABA dipole interactions in a more in-depth matter. The empirical factor $g_1$ calculated using Eq. (7) accounts for the relaxation mechanism for the GABA molecules at 22$^{\circ }C$ and decreased from 0.99 to 0.63 when *c* increased from 0.5 to 5.5 m. $g_1$ values at 37.5$^{\circ }C$ ranged from 0.97–0.67. We obtain

7 $$\begin{aligned} \frac{\hat{\mu }_{c}}{\hat{\mu }_{c\rightarrow 0}} \approx \frac{\sqrt{g_{j=1}}(c)}{\sqrt{g_{j=1}}(c\rightarrow 0)} = \sqrt{g_{j=1}}(c). \end{aligned}$$

When *c* $\rightarrow$ 0, $g_1$ = 1, because in the limit of infinite dilution, there is no correlation between the GABA dipoles. The correlation factors we found were all lower than the value 1, which indicates the antiparallel orientation of the dipoles of GABA. The $g_1$ value decreases as the GABA concentration increases, which suggests that the antiparallel molecular dipole correlation gets stronger when the GABA concentration increases. As the antiparallel molecular dipole correlation increases, there are more dipole vectors with opposite directions that cancel each other out. Comparing the two cases, we notice that the $g_1$ values are overall higher at 37.5$^{\circ }C$ than the $g_1$ values at 22$^{\circ }C$, suggesting that there are less antiparallel correlations between the GABA dipoles as the temperature increases.

At low GABA concentrations, the $g_1$ values are very close to 1, suggesting that there are only slight correlations between the GABA dipoles, as the dipoles are far away from each other. Whereas, at high concentrations, the $g_1$ values differ significantly from 1, which indicates a much stronger dipole correlation, as the GABA dipoles are closer to each other.

### Relative contribution of water

**Figure 5:**
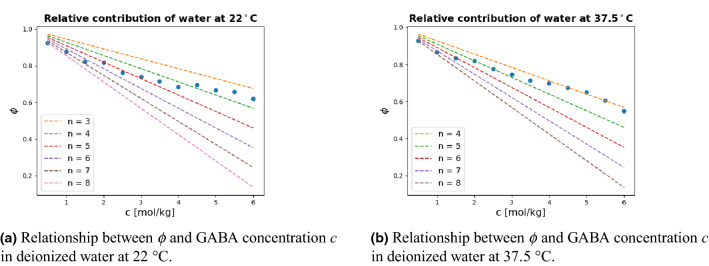
Relative contribution of the water molecules as a function of the GABA concentration *c*, in deionized water at 22$^{\circ }C$ and 37.5$^{\circ }C$. Each straight line represents a prediction of the number of water molecules *n* bound to each GABA molecule.

The relative contribution of water molecules to the total dielectric behavior can be represented with $\phi$ [dimensionless]. Figure 5 shows the dependence of $\phi$ on the GABA concentration. The experimental data points $\phi$ were calculated by using Eq. (8) as

8 $$\begin{aligned} \Delta \epsilon _1' = \epsilon '- \phi \epsilon '_{\text {w}}. \end{aligned}$$

where the $\epsilon '_{\text {w}}$ is the dielectric constant of pure water. The dependence of $\phi$ on the concentration of GABA is relevant to the total number of water molecules that are tightly bound each of the GABA molecules (*n*)^4^. The $\Delta \epsilon _1'$ is the relaxation strength of GABA. The experimentally obtained $\epsilon '_{\text {w}}$ value for GABA in water at 22$^{\circ }C$ and 37.5$^{\circ }C$ was 79.5 and 73.6, respectively. Moreover, the dashed line $\phi$ values were estimated using Eq. (9)

9 $$\begin{aligned} \phi =\frac{1-V_{\text {m}} c}{1+0.5V_{\text {m}} c} - \frac{nc}{c_{\text {w}}} \end{aligned}$$

where $V_{\text {m}}$ represents the volume of a single GABA molecule. The water concentration $c_{\text {w}}$ is 55.6 mol/kg. *c* is the GABA concentration. The dependence of $\phi$ on GABA concentration *c* is sensitive to the contribution of the GABA molecule volume and the total number of tightly bound water molecules *n* for each of the GABA molecules. As Fig. 5a and b present, at low GABA concentrations, there are eight water molecules that are tightly bound to each of the GABA molecules. With increasing concentration, the total number of the bound water molecules decreases to around four. This implies that when the proportion of GABA molecules exceeds a certain degree, the temperature dependence of the number of bound water molecules gets very limited. This is probably due to the antiparallel arrangement of the GABA dipoles and the effect of the GABA molecules getting closer to each other leading to reduced space for water molecules.

At the highest concentration investigated, the molal ratio of the solute GABA molecules and the solvent water molecules is more than 1/10 for the two cases with deionized water as solvent. This means that the number of water molecules is ten times higher than that of the GABA molecules. If the GABA molecules are independently and homogeneously hydrated by the water molecules, then theoretically each of the GABA molecules should have around ten water molecules surrounding it. However, at 6.0 m, a tightly hydrated water number of around four was found. Firstly, this indicates that each of the GABA molecules is most likely not independently hydrated, there is rather a close interaction between the GABA-GABA dipoles when GABA molecules get fairly close to each other, which in turn confirms the theory regarding the strong antiparallel arrangement of the GABA molecules at high concentrations. Secondly, this indicates that there must be water molecules that are not bound with the GABA molecules, even at high concentrations. We suggest that the hydrogen bonding between the GABA molecules and the water molecules did not play a crucial role in the considerably increased relaxation times at high GABA concentrations and the insignificantly different number of water molecules between the two examined cases. However, the hydrogen bonding might give a small contribution in hindering the rotational motion of the GABA molecules.

### Viscosity dependence

**Figure 6:**
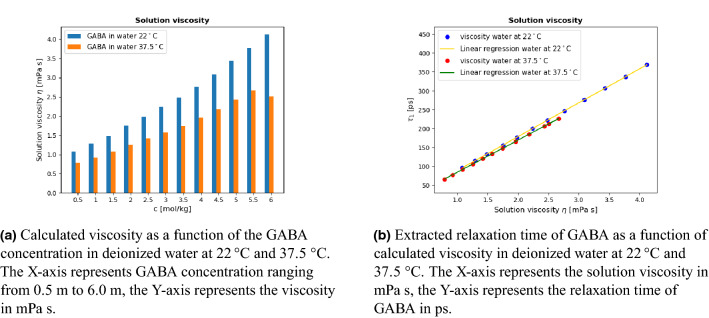
(**a**) Shows the calculated viscosity for different GABA concentrations, and (**b**) shows the correlation between GABA solution viscosity and the GABA relaxation time. The GABA relaxation time $\tau _1$ used was the extracted relaxation time at a concentration range of 0.5 m to 6.0 m.

The relaxation mechanisms of molecules can be affected by the solution’s viscosity. The behavior of the solutions’ viscosity depends on the nature of both water and GABA molecules. Figure 6a shows the viscosity of the GABA solutions as a function of GABA concentration, calculated using Eq. (10):

10 $$\begin{aligned} \eta = \frac{\tau _1 k_{\text {B}}T}{3V_{\text {m}}}. \end{aligned}$$

here $V_{\text {m}}$ is the volume of the molecule^10^ ($V_{\text {m}}$ = 121.60$\times 10^{-30}$ ${\text {m}}^3$), $\tau$ is the relaxation time, $k_{\text {B}}$ is Boltzmann’s constant and *T* is the temperature in Kelvins.

Comparing the viscosity for GABA in deionized water at 22$^{\circ }C$ and 37.5$^{\circ }C$, the viscosity at 22$^{\circ }C$ is much higher than that of 37.5$^{\circ }C$, which indicates that the viscosity decreases with increasing temperature. We suggest that the observed increased relaxation times are highly related to the viscosity of the GABA solution. Figure 6b shows that the relaxation times are proportional to the solution’s viscosity. Higher viscosity will most likely lead to a longer rotational time for the molecules, and thus result in a significant increase of the relaxation time.

However, there are some assumptions that may not hold in Eq. (10), it only depends on the relaxation time and the volume of a GABA molecule at an infinite dilution limit, and it does not account for the change in GABA volume caused by the interactions between GABA molecules at high concentration. The value used for a single GABA molecule volume was obtained from the experiments performed by Sirimulla et al.^10^. Aside from the possible experimental errors, the different temperatures used may be a source of error. The temperature may have a certain effect on the conformation of the GABA molecule thus affecting the GABA molecule volume. The value of GABA molecule volume used in calculating the viscosity has likely introduced some uncertainties to our obtained viscosity values, especially at 37.5$^{\circ }C$.

## Conclusions

A significant behavioral change in complex permittivity of high GABA concentration solutions at physiological temperature was observed. We hypothesize that there is a phase transition in the GABA molecules, which might indicate a significant change in GABA properties at the physiological temperature. The GABA molecules exhibit a more folded topology with decreasing relaxation time as we approach the physiological temperature. This might have an effect on the molecular recognition mechanism. GABA relaxation time is more dependent on the solution viscosity, rather than the dipole moment. The antiparallel correlation between GABA-GABA molecules increases with the increasing concentration and does not seem to be very dependent on temperature. Based on the relative contribution of the water molecules, we found that the GABA molecules are not independently hydrated and even at very high concentrations, as there are free water molecules that are not in any contact with the GABA molecules. At high GABA concentrations, the number of tightly bound water molecules on each of the GABA molecules were approximately four. This implies that when the proportion of the GABA molecules exceeds a certain degree, the effect of the temperature and the solvent dependency of the number of hydrated water molecules gets very limited.

## Methods

### Instrumentation

The DRS was performed with the open end coaxial probe (OCP) method^25^. We used the advanced implementation of the OCP probes and solvers of SPEAG (Schmid & Partner Engineering AG, Switzerland), namely DAK 1.2E: 5–67 GHz and DAK 3.5: 200 MHz–20 GHz probes. Two different vector network analyzers (VNA) were used, the R140 (Copper Mountain Technologies) for the frequency range 85 MHz–14 GHz and the ZVA67 (Rohde & Schwarz) for the frequency range 10 MHz–67 GHz. Combining the probes and the VNAs, our final measurement frequency range was 200 MHz to 67 GHz. The standard 3-point calibration was performed prior to each measurement session, namely, “Open”, “Short”, and “Load” using de-ionized water^26,27^. To estimate the uncertainty of the measurement (Table 1 in the supplementary material) we used the methodology described by Gregory and Clarke^25^ that includes estimates of possible systematic errors due to design, calibration uncertainties, temperature differences between the calibration and measurements, and VNA noise.

The extraction of the complex dielectric parameters from the complex reflection coefficient, $S_{11}$, is based on a full-wave analysis of Maxwell’s equations in cylindrical geometry for semi-infinite samples. The influence of possible extra reflection from the sample boundaries of the finite configurations was examined by moving the sample with respect to the open end coaxial probe. No change was observed in the measured $S_{11}$ within the measurement uncertainty budget.

### Sample preparation

GABA (purity $\ge$ 99%) was purchased from Sigma-Aldrich. GABA samples in the interval of 0.5 molal to 6.0 molal (m) were prepared. Samples were made using 12 equal 0.5 m steps (GABA weight: 25.78 g ± 0.02 g) from 0.5 to 6.0 m and stored in suitable individual flasks. The flask with 0.5 m of GABA was mixed with 500 ml of deionized water (type II) to create 0.5 m GABA solution sample. To make GABA samples of higher concentrations, we used the 0.5 m GABA solution where we sequentially added $M_{\text {0.5 m}}$ = 25.78 g quantity of GABA powder, to increase the concentration from 0.5 to 6.0 m. The above described method was repeated once for GABA in water at 22 °C and once for 37.5 °C.

## Supplementary Information

Supplementary Table 1.

## References

[1] Dyke K (2017). Comparing GABA-dependent physiological measures of inhibition with proton magnetic resonance spectroscopy measurement of GABA using ultra-high-field MRI. NeuroImage.

[2] Lombardi A, Jedlicka P, Luhmann HJ, Kilb W (2019). Interactions between membrane resistance, GABA-A receptor properties, bicarbonate dynamics and CI-transport shape activity-dependent changes of intracellular CI-concentration. Int. J. Mol. Sci..

[3] Sharma B, Chandra A (2018). On the issue of closed versus open forms of gamma-aminobutyric acid (GABA) in water: Ab initio molecular dynamics and metadynamics studies. J. Chem. Phys..

[4] Shikata T, Hashimoto K (2003). Dielectric features of neurotransmitters, γ-aminobutyric acid and l-glutamate, for molecular recognition by receptors. J. Phys. Chem. B.

[5] Wong CGT, Bottiglieri T, Snead OC (2003). GABA, γ-hydroxybutyric acid, and neurological disease. Ann. Neurol..

[6] Cheng B (2018). The γ-aminobutyric acid (GABA) alleviates salt stress damage during seeds germination of white clover associated with Na+/K+ transportation, dehydrins accumulation, and stress-related genes expression in white clover. Int. J. Mol. Sci..

[7] Ottosson N, Pastorczak M, van der Post ST, Bakker HJ (2014). Conformation of the neurotransmitter γ-aminobutyric acid in liquid water. Phys. Chem. Chem. Phys..

[8] Kaatze U, Bieler H, Pottel R (1985). Dielectric spectroscopy on aqueous solutions of some zwitterionic amino acids. J. Mol. Liq..

[9] Rodríguez-Arteche I, Cerveny S, Alegría Á, Colmenero J (2012). Dielectric spectroscopy in the GHz region on fully hydrated zwitterionic amino acids. Phys. Chem. Chem. Phys. PCCP.

[10] Sirimulla S, Lerma M, Herndon WC (2010). Prediction of partial molar volumes of amino acids and small peptides: Counting atoms versus topological indices. J. Chem. Inform. Model..

[11] Levy E, Cerveny S, Ermolina I, Puzenko A, Feldman Y (2014). Dielectric spectra broadening as a signature for dipole-matrix interaction. IV. Water in amino acids solutions. J. Chem. Phys..

[12] Aparicio S, Alcalde R (2010). On the structure of liquid methyl salicylate: The role of intramolecular hydrogen bonding. Eur. J. Chem..

[13] Senthilkumar P (2018). Dielectric dispersion, relaxation and molecular interaction of pyrazine binary mixtures. J. Phys. Commun..

[14] Liu, J. Z. Molecular gravity and phase transition. *Stanford University, California, USA.* https://cs.stanford.edu/people/zjl/state.html (2019).

[15] Liu, J. Z. The forces causing phase transition of matter. *Stanford University, California, USA. *https://www-cs.stanford.edu/people/zjl/pdf/state0.pdf (2019).

[16] Dote JL, Kivelson D, Schwartz RN (1981). A molecular quasi-hydrodynamic free-space model for molecular rotational relaxation in liquids. J. Phys. Chem..

[17] Cavell EAS, Knight PC, Sheikh MA (1971). Dielectric relaxation in non aqueous solutions. Part 2-solutions of tri(n-butyl)ammonium picrate and iodide in polar solvents. Trans. Faraday Soc..

[18] Böttcher CJF, Rip A, Bordewijk P, Belle OCV (1973). Theory of Electric Polarization.

[19] Barthel J, Buchner R (1992). Dielectric permittivity and relaxation of electrolyte solutions and their solvents. Chem. Soc. Rev..

[20] Shiraga K (2015). Broadband dielectric spectroscopy of glucose aqueous solution: Analysis of the hydration state and the hydrogen bond network. J. Chem. Phys..

[21] Kirkwood JG (1939). The dielectric polarization of polar liquids. J. Chem. Phys..

[22] Samanta N, Mahanta DD, Choudhury S, Barman A, Mitra RK (2017). Collective hydration dynamics in some amino acid solutions: A combined GHz–THz spectroscopic study. J. Chem. Phys..

[23] Abeyrathne CD, Halgamuge MN, Farrell PM, Skafidas E (2014). Dielectric properties of liquid phase molecular clusters using the external field method: Molecular dynamics study. Phys. Chem. Chem. Phys..

[24] Odai K, Sugimoto T, Hatakeyama D, Kubo M, Ito E (2001). A theoretical study of electronic and structural states of neurotransmitters: Gamma-aminobutyric acid and glutamic acid. J. Biochem..

[25] Gregory AP, Clarke RN (2007). Dielectric metrology with coaxial sensors. Meas. Sci. Technol..

[26] Kaatze U (1989). Complex permittivity of water as a function of frequency and temperature. J. Chem. Eng. Data.

[27] Ellison WJ (2007). Permittivity of pure water, at standard atmospheric pressure, over the frequency range 0–25THz and the temperature range 0–100 °C. J. Phys. Chem. Ref. Data.

