# Supplementary material for: Temperature dependence of the microwave dielectric properties of γ-aminobutyric acid
Source: Sci Rep. 2021 Sep 10;11:18082. doi: 10.1038/s41598-021-97178-7 (PMC8433407; doi:10.1038/s41598-021-97178-7)
Supplement: Supplementary file 1 — Supplementary Table 1. [file 41598_2021_97178_MOESM1_ESM.docx]

|  | DAK-3.5 | |
| --- | --- | --- |
| Freq (GHz) | Δε (rel.) | Δσ (rel.) |
| 0.2 | 1.7% | 2.7% |
| 0.3 | 1.7% | 2.7% |
| 0.5 | 1.7% | 2.7% |
| 1 | 1.7% | 2.7% |
| 2 | 1.7% | 2.7% |
| 3 | 1.7% | 2.7% |
| 5 | 2.3% | 3.0% |
| 6 | 2.3% | 3.0% |
| 10 | 3.5% | 3.0% |
| 15 | 3.5% | 3.0% |
| 20 | 3.5% | 3.0% |
